# Association between familial aggregation of chronic kidney disease and its incidence and progression

**DOI:** 10.1038/s41598-023-32362-5

**Published:** 2023-03-29

**Authors:** Jae Young Kim, Sung-youn Chun, Hyunsun Lim, Tae Ik Chang

**Affiliations:** 1 Department of Internal Medicine, National Health Insurance Service Ilsan Hospital, 100 Ilsan-ro, Ilsandong-gu, Goyang-si, Gyeonggi-do 10444 Republic of Korea; 2 Department of Internal Medicine, Institute of Kidney Disease Research, Yonsei University College of Medicine, Seoul, Republic of Korea; 3 Research and Analysis Team, National Health Insurance Service Ilsan Hospital, Goyang-si, Gyeonggi-do Republic of Korea

**Keywords:** Nephrology, Risk factors

## Abstract

This study aimed to examine the association between familial aggregation of chronic kidney disease (CKD) and risk of CKD development and its progression. This nationwide family study comprised 881,453 cases with newly diagnosed CKD between 2004 and 2017 and 881,453 controls without CKD matched by age and sex, using data from the Korean National Health Insurance Service with linkage to the family tree database. The risks of CKD development and disease progression, defined as an incident end-stage renal disease (ESRD), were evaluated. The presence of any affected family member with CKD was associated with a significantly higher risk of CKD with adjusted ORs (95% CI) of 1.42 (1.38–1.45), 1.50 (1.46–1.55), 1.70 (1.64–1.77), and 1.30 (1.27–1.33) for individuals with affected parents, offspring, siblings, and spouses, respectively. In Cox models conducted on patients with predialysis CKD, risk of incident ESRD was significantly higher in those with affected family members with ESRD. The corresponding HRs (95% CI) were 1.10 (1.05–1.15), 1.38 (1.32–1.46), 1.57 (1.49–1.65), and 1.14 (1.08–1.19) for individuals listed above, respectively. Familial aggregation of CKD was strongly associated with a higher risk of CKD development and disease progression to ESRD.

## Introduction

Chronic kidney disease (CKD) is a growing public health problem not only in South Korea but also worldwide^1,2^. Patients with CKD have a substantially higher risk of cardiovascular disease and mortality even in the early stages of CKD^3–5^. While patients with CKD are five to ten times more likely to die than progress to end-stage renal disease (ESRD), those who survive may ultimately require dialysis treatment or kidney transplantation^6^. These interventions put an exorbitant economic burden on many countries, cost billions of dollars to treat patients with ESRD, and incur substantial financial costs in preventing CKD and its complications^7^.

Family health history has become increasingly recognized as the most useful tool for risk assessment of common chronic diseases^8^. A growing body of literature suggests that individuals with an affected first-degree relative have a higher risk of various cancers^9^, stroke^10^, type 2 diabetes^11^, and cardiovascular diseases^12,13^. Furthermore, associations between family history and kidney diseases have also been reported, but these studies have largely focused on some Mendelian disorders, such as polycystic kidney disease and Alport syndrome. While the causes of CKD are diverse, there is a paucity of large population-based cohort studies that have examined the familiar contributions to the broader spectrum of CKD to date. Furthermore, there are significant shortcomings in these few studies that examined whether CKD aggregates within family, which result from small sample size, data acquired via hospital records or registries, information derived from questionnaires, and definition of study variables focused mainly on its later stage (i.e., ESRD)^14–19^. Therefore, this study aimed to determine the association between family history of CKD and risk of incident CKD and its progression to ESRD in a large nationwide population-based cohort using data from the Korean National Health Insurance Service (NHIS) database linked to the family tree database to better inform the field.

## Materials and methods

### Data source and study population

Data were obtained from the Korean NHIS database linked to the nationwide family tree database. Since the NHIS covers compulsory health insurance for all citizens in Korea as a single-payer national health system, all medical records of covered inpatient and outpatient visits are centralized in the NHIS database^20,21^. The family tree database provides details on family relationships and degree of kinship (grandparents, parents, offspring, full siblings, and spouses) for the entire population, which was created using a new family code system, health insurance eligibility, and resident register data. The methods for constructing the database have been described previously, in which parents and grandparents are matched for more than 95% of those who were born between 2010 and 2017^22^.

To construct the study population of this nationwide case–control study, we first identified all 983,736 adult patients (age ≥ 18 years) having a diagnosis of CKD recorded between 1 January 2004 and 31 December 2017. To restrict the cohort with newly diagnosed patients with CKD, 93,506 patients who had claims for CKD during a washout period of two years from 1 January 2002 to 31 December 2003 were excluded. Ascertainment of CKD was based on *the International Statistical Classification of Disease and Related Health Problems, Tenth Revision* (ICD-10) code of N18, and index date was defined as the date of the first diagnosis of CKD. Furthermore, to minimize errors in the estimation of familial risk associated with the more common causes of CKD, 8777 patients with claims for hereditary kidney diseases such as polycystic kidney disease (ICD-10 codes Q61.1 and Q61.2; *n* = 7140), medullary cystic kidney disease (ICD-10 code Q61.5; *n* = 248), Fabry disease (ICD-10 codes E75.2 and N08.4; *n* = 1028), and Alport syndrome (ICD-10 code Q87.8; *n* = 361) were excluded. After exclusion, a total of 881,453 patients with incident CKD were included in the study. For each case, we randomly assigned index date to controls as the same date of the matched cases. And then we matched each case of patients with one control by age and sex at the time of index date, who did not have a diagnosis of CKD from 1 January 2002 to a randomly assigned index date drawn from the corresponding dates in the CKD cases. Therefore, the final study population comprised 1,762,906 participants, which included 881,453 cases with CKD and 881,453 matched controls without CKD (Fig. S1). This study complied with the Declaration of Helsinki and was approved by the Institutional Review Board of NHIS Ilsan Hospital, which waived the requirement for informed consent due to the use of deidentified data.

### Data collection and covariables

We considered age, sex, residential area, income level, and comorbidities such as hypertension, diabetes, ischemic heart disease, cerebrovascular disease, and dyslipidemia to be potential confounders or to potentially affect familial associations. Thus, they were included as covariables^23^. Baseline data on sociodemographic information such as age, sex, residential area, and income level were collected before the index date. Comorbidities (e.g., hypertension (I10 ~ 13, I15), diabetes (E10 ~ 14), ischemic heart disease (I20 ~ 25), cerebrovascular disease (I60 ~ 69), and dyslipidemia (E78.0 ~ 78.5)) were assessed using the ICD-10 coding algorithms, which were ascertained by the presence of at least two or more diagnostic codes up to two years before the index date. The presence of affected family members with CKD, along with or without ESRD, was assessed using the nationwide claims database in conjunction with the family tree database at the time of the index date.

### Exposure and outcome ascertainment

The exposure of interest was a familial aggregation of CKD. A family was defined as a group of individuals related to each other by blood or by at least one common blood relative, including first-degree relatives (i.e., parents and offspring), full siblings, and spouses. The outcomes of interest were incident CKD and CKD progression, with CKD progression being defined as an incident ESRD. ESRD was defined as receipt of long-term dialysis or a kidney transplant, identified by specific insurance codes (called V code) or dialysis-related intervention codes^24^. NHIS provides special insurance benefits for patients with ESRD who receive a kidney transplant or require maintenance dialysis for a minimum of 3-month duration. Once a patient has a specific code related to ESRD (e.g., V001 for hemodialysis, V003 for peritoneal dialysis, and V005 for kidney transplant), it is carried forward in medical records and claims created for that patient. Therefore, ESRD diagnoses based on claims are considered reliable.

### Statistical analysis

Multivariable logistic regression models with adjustment for age, sex, residential area, income level, and comorbidities such as hypertension, diabetes, ischemic heart disease, cerebrovascular disease, and dyslipidemia were used to examine the association between having an affected family member with CKD and the risk of incident CKD. The risk of CKD was expressed as odds ratios (OR) with 95% confidence intervals (CI).

Next, Cox proportional hazards models with the presence of an affected individual with ESRD as a predictor were conducted to assess the risk of an incident ESRD among patients with predialysis CKD, adjusting for all covariables that were used to construct the multivariable logistic regression models as above. For this analysis, patients with a prior diagnosis of ESRD at any time before the index date were excluded. Follow-up began on the index date and continued until the occurrence of ESRD, death, or 31 December 2017 (study end date), whichever came first. The risk of ESRD was represented as hazard ratios (HR) with 95% CI. To further address the potential influence of unmeasured confounding on the analyses, we performed additional sensitivity analyses using the E-value methodology. The E-value represents the minimum magnitude of association required between unmeasured confounder and both the exposure and outcome, conditional on measured covariables, to fully attenuate the observed exposure-outcome relationship^25^. Each E-value was calculated using a publicly available online calculator^26^.

All models were explored for individuals with an affected first-degree relative of any kinship and for individual kinship (e.g., parents, offspring, and full siblings) in the overall cohort and subpopulation stratified by sex. Furthermore, spouses were also used as controls to account for contributions from shared environmental factors to phenotypic variance. Data from descriptive analyses were summarized using mean (standard deviation (SD)) or numbers (proportions), as appropriate. All analyses were performed using SAS version 9.4 (SAS Institute Inc., Cary, NC).

## Results

### Baseline characteristics of study population

A total of 1,762,906 participants who met the eligibility criteria were included in the study. The baseline characteristics of the participants are shown in Table 1. The mean age of the study participants was 64.2 (SD, 16.0) years. Among them, 57.1% were male, 57.9% had hypertension, and 33.6% had diabetes. In the overall cohort, 7.7% and 3.1% of participants had at least one affected family member with CKD or ESRD, respectively: 35,455 (2.0%) with affected parents, 32,841 (1.9%) with an affected offspring, 18,609 (1.1%) with an affected sibling, and 54,056 (3.1%) with an affected spouse for CKD and 13,687 (0.8%) with affected parents, 14,985 (0.9%) with an affected offspring, 8750 (0.5%) with an affected sibling, and 19,110 (1.1%) with an affected spouse for ESRD.

**Table 1:** Baseline characteristics of the study participants.

	Overall	Study participants	
		Cases	Matched controls
Characteristics	(n = 1,762,906)	(n = 881,453)	(n = 881,453)
Age, years	64.2 (16.0)	64.2 (16.0)	64.2 (16.0)
Age intervals			
< 40 years	142,935 (8.1)	71,467 (8.1)	71,468 (8.1)
40–49 years	172,707 (9.8)	86,354 (9.8)	86,353 (9.8)
50–59 years	293,100 (16.6)	146,550 (16.6)	146,550 (16.6)
60–69 years	386,470 (21.9)	193,234 (21.9)	193,236 (21.9)
70–79 years	476,544 (27.1)	238,246 (27.1)	238,298 (27.1)
≥ 80 years	291,150 (16.5)	145,602 (16.5)	145,548 (16.5)
Sex			
Men	1,006,266 (57.1)	503,177 (57.1)	503,089 (57.1)
Women	756,640 (42.9)	378,276 (42.9)	378,364 (42.9)
Residential area			
Metropolitan	717,193 (40.7)	369,612 (41.9)	347,581 (39.4)
Large city	429,804 (24.4)	213,604 (24.3)	216,200 (24.5)
Small city and rural area	615,909 (34.9)	298,237 (33.8)	317,672 (36.1)
Income quartiles			
First quartile (lowest)	420,551 (23.9)	224,921 (25.5)	195,630 (22.2)
Second quartile	329,474 (18.7)	162,918 (18.5)	166,556 (18.9)
Third quartile	432,303 (24.5)	210,633 (23.9)	221,670 (25.1)
Fourth quartile (highest)	580,578 (32.9)	282,981 (32.1)	297,597 (33.8)
Comorbidities			
Hypertension	1,021,250 (57.9)	658,096 (74.7)	363,154 (41.2)
Diabetes	592,795 (33.6)	426,989 (48.4)	165,806 (18.8)
Ischemic heart disease	288,602 (16.4)	206,601 (23.4)	82,001 (9.3)
Cerebrovascular disease	254,739 (14.5)	177,895 (20.2)	76,844 (8.7)
Dyslipidemia	681,790 (38.7)	458,702 (52.0)	223,088 (25.3)
Affected family member			
CKD	135,353 (7.7)	80,666 (9.2)	54,687 (6.2)
ESRD	55,334 (3.1)	34,299 (3.9)	21,035 (2.4)

Data are presented as means (standard deviation) or numbers (percentages).

*CKD* chronic kidney disease, *ESRD* end-stage renal disease.

Overall, age, sex, residential area, and income level were generally similar across the groups, but comorbid conditions were more prevalent in patients with CKD. Additionally, cases were more likely to have an affected family member with CKD (9.2% vs. 6.2%) or ESRD (3.9% vs. 2.4%) than age- and sex-matched controls.

### Risks of CKD in individuals with affected relatives with CKD

**Figure 1:**
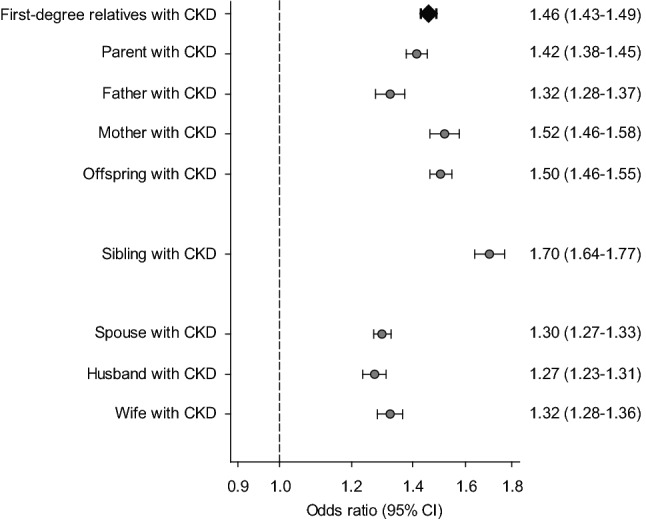
Risks of CKD in individuals having affected relatives with CKD. All models were adjusted for age, sex, residential area, income level, and comorbidities such as hypertension, diabetes, ischemic heart disease, cerebrovascular disease, and dyslipidemia. CKD, chronic kidney disease; CI, confidence interval.

In logistic regression models adjusted for sociodemographic data and comorbidities, the presence of any affected family member with CKD was associated with a significantly higher risk of CKD (Table 2 and Fig. 1). Overall, adjusted OR (95% CI) for individuals with affected first-degree relatives with CKD was 1.46 (1.43–1.49): specifically, 1.42 (1.38–1.45) for individuals with affected parents and 1.50 (1.46–1.55) for individuals with affected offspring, respectively. Additionally, having an affected sibling or spouse was associated with a higher risk of CKD, with OR (95% CI) of 1.70 (1.64–1.77) and 1.30 (1.27–1.33) in individuals with affected siblings and spouses, respectively. Of note, further subgroup analyses confirmed the strong and consistent association between familial aggregation of CKD and risk of CKD in both men and women (Fig. S2).

**Table 2:** Association between familial aggregation of CKD and risk of CKD.

Type of affected family member	Study participants, number (%)		OR (95% CI)
	Cases (n = 881,453)	Matched controls (n = 881,453)	
First-degree relatives	41,441 (4.7)	26,704 (3.0)	1.46 (1.43–1.49)
Parents	21,413 (2.4)	14,042 (1.6)	1.42 (1.38–1.45)
Father	10,637 (1.2)	7464 (0.9)	1.32 (1.28–1.37)
Mother	11,645 (1.3)	6960 (0.8)	1.52 (1.46–1.58)
Offspring	20,142 (2.3)	12,699 (1.4)	1.50 (1.46–1.55)
Sibling	12,108 (1.4)	6501 (0.7)	1.70 (1.64–1.77)
Spouse	30,920 (3.5)	23,136 (2.6)	1.30 (1.27–1.33)
Husband	15,992 (1.8)	12,116 (1.4)	1.27 (1.23–1.31)
Wife	14,928 (1.7)	11,020 (1.3)	1.32 (1.28–1.36)

All models were adjusted for age, sex, residential area, income level, and comorbidities such as hypertension, diabetes, ischemic heart disease, cerebrovascular disease, and dyslipidemia.

*CKD* chronic kidney disease, *OR* odds ratio, *CI* confidence interval.

### Risks of ESRD in patients with predialysis CKD with affected relatives with ESRD

**Figure 2:**
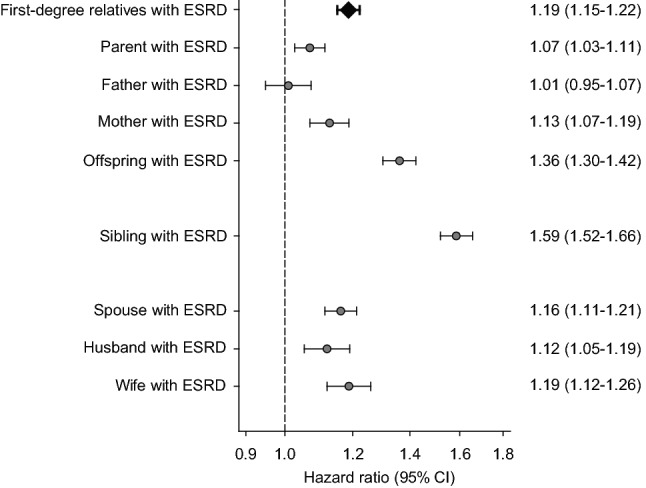
Risks of ESRD in patients with predialysis CKD having affected relatives with ESRD. All models were adjusted for age, sex, residential area, income level, and comorbidities such as hypertension, diabetes, ischemic heart disease, cerebrovascular disease, and dyslipidemia. CKD, chronic kidney disease; CI, confidence interval; ESRD, end-stage renal disease.

In this study, we aimed to examine the association between familial aggregation of ESRD and the risk of incident ESRD among patients with predialysis CKD. For this analysis, of the 881,453 patients with CKD, 66,318 patients with a prior diagnosis of ESRD were excluded, and the analysis was conducted on a total of 815,135 patients with non-dialysis dependent CKD, among whom 31,512 (3.9%) individuals had at least one affected family member with ESRD. During a mean follow-up of 3.9 (SD, 3.6) years (3,207,497 person-years of follow-up), a total of 126,483 (15.5%) incident ESRD events occurred: 6512 and 119,971 events in patients with and without an affected family member with ESRD, respectively. In Cox regression models, the risks of incident ESRD were significantly higher in individuals with affected first-degree relatives, parents, offspring, siblings, and spouses with a corresponding HR (95% CI) of 1.22 (1.17–1.26), 1.10 (1.05–1.15), 1.38 (1.32–1.46), 1.57 (1.49–1.65), and 1.14 (1.08–1.19), respectively. Although these higher observed risks were markedly attenuated in individuals with an affected father, the small sample size in this group makes this association less reliable (Table 3 and Fig. 2). Similar to findings in the overall cohort, individuals with any affected family member with ESRD tended to have a higher risk of ESRD across subgroups stratified by sex, but the associations of affected parents were much attenuated in female patients with CKD (Fig. S3).

**Table 3:** Association between familial aggregation of ESRD and risk of incident ESRD.

Type of affected family member	Event number/patient number (%)		HR (95% CI)
	With affected family member	Without affected family member	
First-degree relatives	3447/16,588 (20.8)	123,036/798,547 (15.4)	1.22 (1.17–1.26)
Parents	1858/8235 (22.6)	124,625/806,900 (15.4)	1.10 (1.05–1.15)
Father	795/3797 (20.9)	125,688/811,338 (15.5)	1.03 (0.96–1.11)
Mother	1099/4555 (24.1)	125,384/810,580 (15.5)	1.15 (1.09–1.23)
Offspring	1593/8371 (19.0)	124,890/806,764 (15.5)	1.38 (1.32–1.46)
Sibling	1613/5425 (29.7)	124,870/809,710 (15.4)	1.57 (1.49–1.65)
Spouse	1687/10,266 (16.4)	124,796/804,869 (15.5)	1.14 (1.08–1.19)
Husband	828/5110 (16.2)	51,572/345,210 (14.9)	1.10 (1.03–1.18)
Wife	859/5156 (16.7)	73,224/459,659 (15.9)	1.15 (1.07–1.23)

All models were adjusted for age, sex, residential area, income level, and comorbidities such as hypertension, diabetes, ischemic heart disease, cerebrovascular disease, and dyslipidemia.

*HR* hazard ratio, *CI* confidence interval, *ESRD* end-stage renal disease.

To further substantiate our findings, we calculated E-values to assess the potential influence of unmeasured confounders on the association between familial aggregation of CKD and its incidence and progression. Given that point estimates and upper CIs of each E-value were seemingly remote beyond those of confounders that were measured, it is less likely that unmeasured confounders exist that can overcome the associations observed in this study (Tables S1 and S2).

## Discussion

This nationwide population-based study of 1.76 million people in South Korea showed a strong familial aggregation of CKD such that individuals with an affected family member with CKD had a higher risk of incident CKD. Furthermore, once CKD had been diagnosed, family history of ESRD was also associated with a significantly higher risk of disease progression to ESRD. Thus, these findings reveal that the family history of kidney disease may be useful to early identify individuals at high risk of CKD and accurately classify patients’ risk of ESRD among patients with CKD.

There has been accumulating evidence that CKD has a familial predisposition. Over 30 years ago, Ferguson et al.^27^ reported that a family history of CKD was associated with a substantially higher risk of ESRD. A year later, Seaquist et al. found that there is a striking concordance of diabetic nephropathy between siblings with type 1 diabetes^28^. Additionally, Lei et al.^29^ showed that the risk of ESRD was significantly higher in individuals with any family history of renal disease, and these associations could not be completely explained by clustering of other known risk factors for ESRD within the family, such as diabetes and hypertension. Notably, these observations have inspired several studies to search for genes contributing to the risk of a wider range of kidney diseases in the twenty-first century. For example, genome-wide association studies have identified many genetic regions associated with renal traits, such as diabetic nephropathy, estimated glomerular filtration rate, and albuminuria^30–35^. As another notable discovery, the polymorphisms in the APOL1 (apolipoprotein L1) gene were found in 2010, which conferred very high risks of hypertensive nephrosclerosis and focal global glomerulosclerosis in a recessive manner^36–38^. Interestingly, the higher prevalence of such a pathogenic APOL1 allele in Black Americans has been recognized as one of the plausible explanations responsible for the higher burden of CKD in this population than in White Americans^39,40^. Recently, the familial risk of CKD and ESRD has also been confirmed in several large observational studies. A cross-sectional, population-based cohort study including 87,849 Taiwanese patients with ESRD found that there was an association between having an affected first-degree relative with ESRD and the development of ESRD with a relative risk of 2.46 (95% CI, 2.32–2.62)^19^. Furthermore, Zhang et al. more recently reported similar findings in European patients with an earlier stage of CKD, including 1,862 CKD cases of 155,911 study participants, noting that the risk of CKD in individuals with an affected first-degree member was three times higher than that in the general population (recurrence risk ratio 3.04, 95% CI 2.26–4.09)^41^. Their study is particularly noble given that most studies examining familial contributions to kidney diseases have largely focused on the advanced stages, ESRD. As an extension of these studies, our study additionally confirmed that individuals with an affected family member with CKD are not only far more likely to develop CKD but also exhibit faster disease progression to ESRD. Interestingly, a significantly higher risk for incident CKD was found in individuals with parents with CKD irrelevant to parent’s sex. However, higher risk for ESRD was observed only in those with affected mothers with ESRD. The reason for this finding is uncertain but we suspect that our database may have intrinsic flaws that may explain the discrepancy. The Korean NHIS database contains information recorded since 2002, and patients with kidney disease who died before 2002 were not included in this database. Eventually, affected individuals with parents who died before 2002 may have been misrepresented as CKD individuals with healthy parents. Furthermore, since the prevalence of CKD is generally higher in women, whereas mortality is higher in men^42,43^, a number of deceased fathers with CKD may not have been accounted in this study. Thus, cautious interpretation is required making a conclusion that fathers are less associated with offspring’s kidney disease compared to mothers. In addition, it is possible that sex of the affected siblings and offspring may have also affected the family aggregation. However, we did not examine the risk regarding sex of relatives due to the absence of information on sex distinction (i.e., brother, sister, son, and daughter) in our dataset. Hence, future studies are needed to ascertain these important issues. Nonetheless, to the best of our knowledge, this is the largest study conducted to date examining more than 1.7 million people in Korea, providing strong statistical power. While the underlying mechanisms responsible for these associations await further investigation, the study findings suggest that a family history of CKD or dialysis is associated with an increased incidence of CKD and disease progression to ESRD and can be used to identify individuals at high risk of both kidney diseases.

A higher risk of kidney disease with an affected family member indicates that CKD is a hereditable condition. However, it is more evident that shared environment and shared genes likely contribute to kidney disease. Assuming that spouses share the family environment but not close genetic similarity with other family members, they have been used to estimate the relative contribution of shared environmental factors to susceptibility to kidney disease^19,41,44^. In this regard, we also found that individuals having affected spouses with CKD were associated with a 30% higher risk of CKD. Likewise, among patients with CKD, those with affected spouses with ESRD were also associated with a 14% increased risk of ESRD. These findings are further supported by the aforementioned studies, which consistently showed that individuals with an affected spouse with CKD or ESRD were associated with a higher burden of each kidney disease. Accordingly, it should be emphasized that both genetic and shared environmental factors might be considered to better understand the complex nature of the familial contribution to kidney disease^45^.

The validity of this study is strengthened by the use of the Korean NHIS database linked to the nationwide family tree database, which contains information on the entire population of South Korea. Given that the awareness of CKD by both patients themselves and other family members is likely to be low (i.e., when compared to awareness of other catastrophic illnesses such as ischemic heart disease, stroke, or malignancy that are often included in family history questionnaires), it seems to be more valid to ascertain an affected family member based on the nationwide family tree database rather than on questionnaire^17^. However, this study has several limitations. First, we ascertained CKD entirely relied on ICD-10 codes due to the lack of relevant laboratory data such as estimated glomerular filtration rate and albuminuria, which might not precisely capture the disease burden. Hence, the study results may underestimate the true prevalence of CKD in this population. Second, residual confounding might still be a limitation as we did not capture complete data on potential risk factors such as blood pressure, obesity, health behaviors (e.g., smoking status), or medications (e.g., use of angiotensin-converting enzyme inhibitors and/or angiotensin-receptor blockers), some of which have been associated with CKD outcomes^23^. Therefore, we could not assume that all measured covariables were sufficient to adjust for all biases. Nonetheless, we tried to address this shortcoming, at least in part by vigorous adjustment for measured covariables such as sociodemographic data and various comorbidities. Furthermore, sensitivity analysis using E-value estimation indicated that contribution of unmeasured confounding to this association was less likely. Third, given the observational nature of our study design, we could not infer the causality of the observed associations between familial aggregation of CKD and disease occurrence and progression. Finally, our findings may not be generalizable to populations outside of South Korea, given the genetic architecture, social factors, environmental exposures, national healthcare policies, and chronic disease burden, including kidney disease, which may be distinct from other countries.

In conclusion, this national family cohort study of the Korean population revealed that a family history of CKD was associated with a significantly higher risk of not only CKD but also ESRD. While more accurate and readily applicable genetic testing is not currently available in routine clinical practice, these intriguing findings provide useful information suggesting that ascertainment of affected family members with CKD or ESRD is useful to early identify individuals at high risk of CKD, which is also valid to predict disease progression in patients with CKD. Hence, it should be emphasized that a detailed family history of kidney diseases should be taken as a part of clinical care to screen and treat high risk individuals in a timely manner.

## Supplementary Information

Supplementary Information.

## Data Availability

All relevant data are within the manuscript and its Supplementary Materials files. Technical appendix and statistical code are available from Dr. Chang upon request (email: kidneyjang@gmail.com).
