# Supplementary material for: Association between familial aggregation of chronic kidney disease and its incidence and progression
Source: Sci Rep. 2023 Mar 29;13:5131. doi: 10.1038/s41598-023-32362-5 (PMC10060248; doi:10.1038/s41598-023-32362-5)
Supplement: Supplementary file 1 — Supplementary Information. [file 41598_2023_32362_MOESM1_ESM.docx]

**Association between Familial Aggregation of Chronic Kidney Disease and Its Incidence and Progression**

Authors:

Jae Young Kim, MD, Sung-youn Chun, PhD, Hyunsun Lim, PhD, and Tae Ik Chang, MD, PhD

**Supplementary Material**

**Supplementary Tables**

**Supplementary Table S1.** E-values for the association between familial aggregation of CKD and risk of CKD.

**Supplementary Table S2.** E-values for the association between familial aggregation of ESRD and risk of incident ESRD.

**Supplementary Figures**

**Supplementary Figure S1.** Flow chart of study cohort construction.

**Supplementary Figure S2.** Subgroup analyses of risks of CKD in individuals having affected relatives with CKD among men (A) and women (B).

**Supplementary Figure S3.** Subgroup analyses of risks of ESRD in patients with predialysis CKD having affected relatives with ESRD among men (A) and women (B).

**Supplementary Table S1.** E-values for the association between familial aggregation of CKD and risk of CKD.

| Type of affected family member | OR (95% CI) | E-value | E-value |
| --- | --- | --- | --- |
|  |  | for point estimate | for CI |
| First-degree relatives | 1.46 (1.43-1.49) | 2.28 | 2.21 |
| Parents | 1.42 (1.38-1.45) | 2.19 | 2.10 |
| Father | 1.32 (1.28-1.37) | 1.97 | 1.88 |
| Mother | 1.52 (1.46-1.58) | 2.41 | 2.28 |
| Offspring | 1.50 (1.46-1.55) | 2.37 | 2.28 |
| Sibling | 1.70 (1.64-1.77) | 2.79 | 2.66 |
| Spouse | 1.30 (1.27-1.33) | 1.92 | 1.86 |
| Husband | 1.27 (1.23-1.31) | 1.86 | 1.76 |
| Wife | 1.32 (1.28-1.36) | 1.97 | 1.88 |

All models were adjusted for age, sex, residential area, income level, and comorbidities such as hypertension, diabetes, ischemic heart disease, cerebrovascular disease, and dyslipidemia. Each E-value for the point estimate or the upper limit of the confidence interval represents the minimum strength of association required between unmeasured confounders and both the exposure and outcome, conditional on measured covariables, to fully attenuate the observed exposure-outcome relationship. CKD, chronic kidney disease; OR, odds ratio; CI, confidence interval.

**Supplementary Table S2.** E-values for the association between familial aggregation of ESRD and risk of incident ESRD.

| Type of affected family member | HR (95% CI) | E-value | E-value |
| --- | --- | --- | --- |
|  |  | for point estimate | for CI |
| First-degree relatives | 1.22 (1.17-1.26) | 1.74 | 1.62 |
| Parents | 1.10 (1.05-1.15) | 1.43 | 1.28 |
| Father | 1.03 (0.96-1.11) | 1.21 | 1.00 |
| Mother | 1.15 (1.09-1.23) | 1.57 | 1.40 |
| Offspring | 1.38 (1.32-1.46) | 2.10 | 1.97 |
| Sibling | 1.57 (1.49-1.65) | 2.52 | 2.34 |
| Spouse | 1.14 (1.08-1.19) | 1.54 | 1.37 |
| Husband | 1.10 (1.03-1.18) | 1.43 | 1.21 |
| Wife | 1.15 (1.07-1.23) | 1.57 | 1.34 |

All models were adjusted for age, sex, residential area, income level, and comorbidities such as hypertension, diabetes, ischemic heart disease, cerebrovascular disease, and dyslipidemia. Each E-value for the point estimate or the upper limit of the confidence interval represents the minimum strength of association required between unmeasured confounders and both the exposure and outcome, conditional on measured covariables, to fully attenuate the observed exposure-outcome relationship. HR, hazard ratio; CI, confidence interval; ESRD, end-stage renal disease.


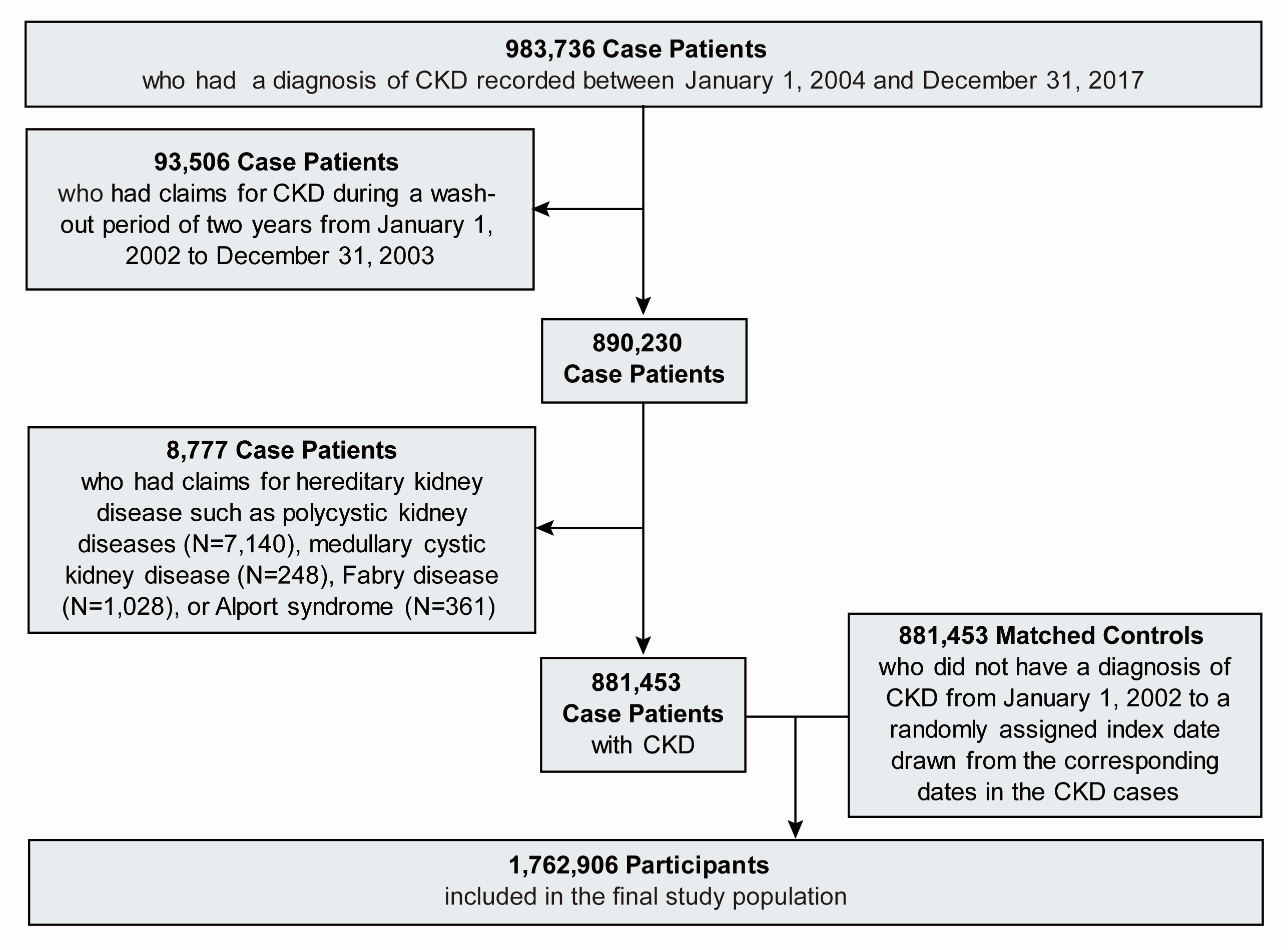


**Supplementary Figure S1.** Flow chart of study cohort construction.


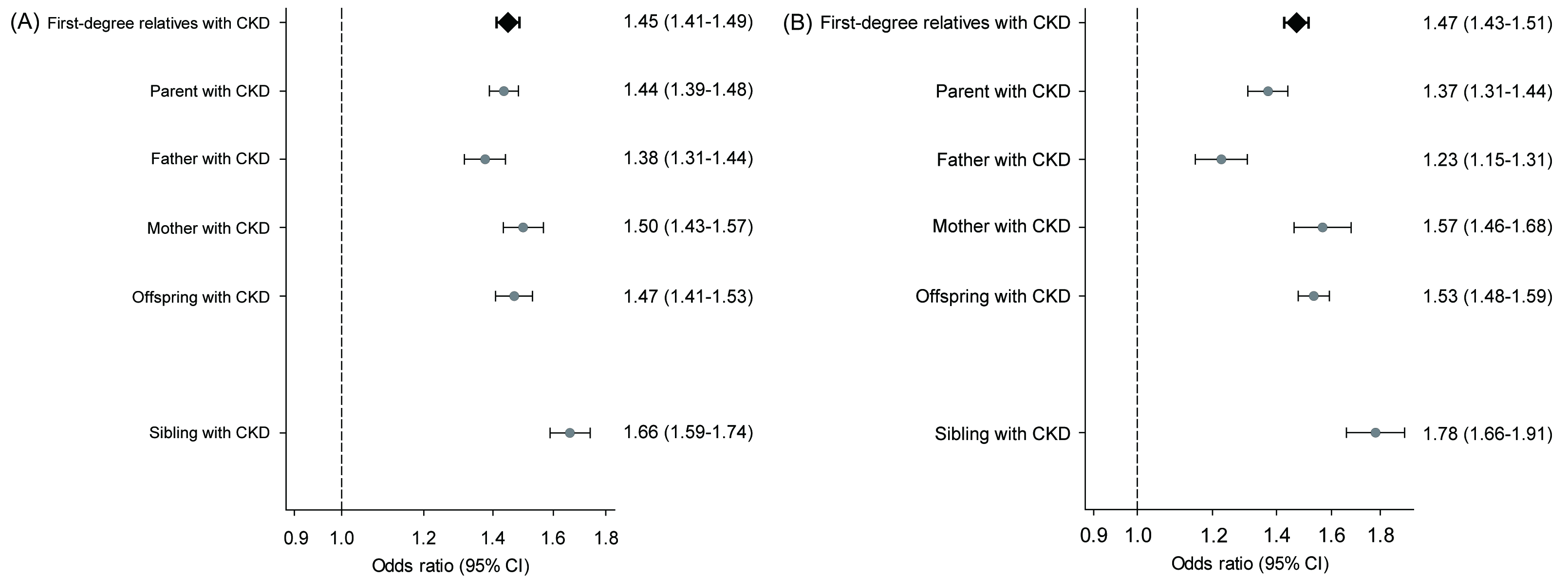


**Supplementary Figure S2.** Subgroup analyses of risks of CKD in individuals having affected relatives with CKD among men (A) and women (B). All models were adjusted for age, sex, residential area, income level, and comorbidities such as hypertension, diabetes, ischemic heart disease, cerebrovascular disease, and dyslipidemia. CKD, chronic kidney disease; CI, confidence interval.


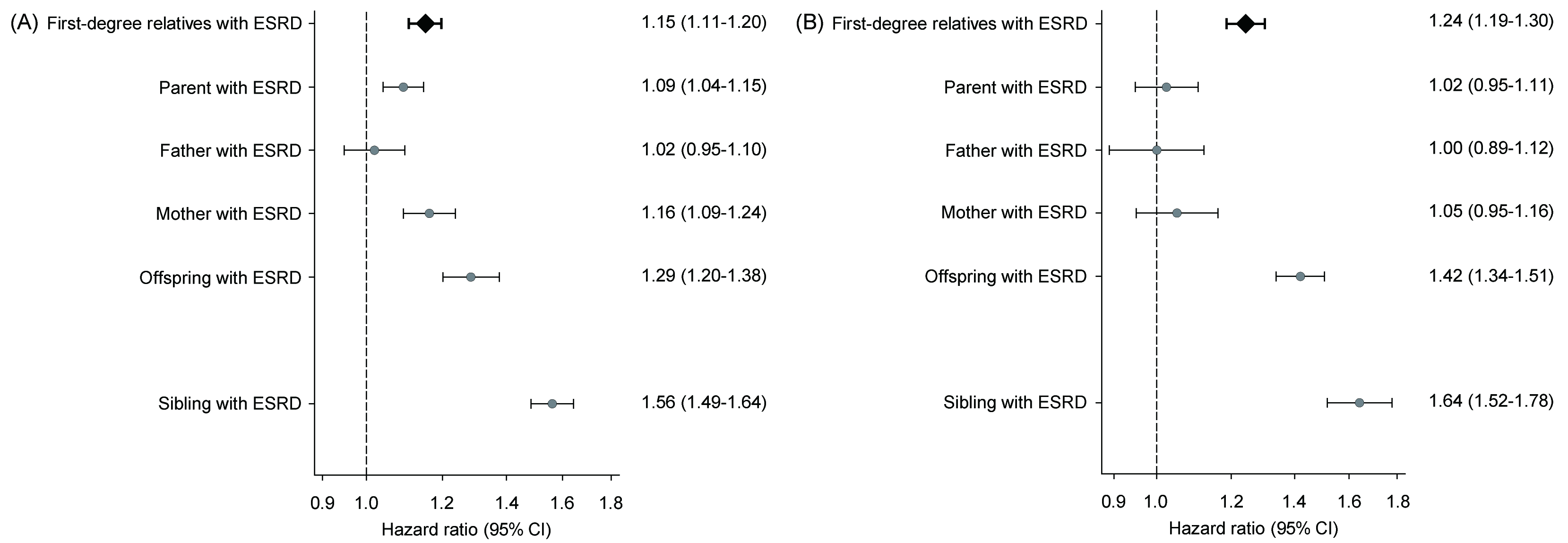


**Supplementary Figure S3.** Subgroup analyses of risks of ESRD in patients with predialysis CKD having affected relatives with ESRD among men (A) and women (B). All models were adjusted for age, sex, residential area, income level, and comorbidities such as hypertension, diabetes, ischemic heart disease, cerebrovascular disease, and dyslipidemia. CKD, chronic kidney disease; CI, confidence interval; ESRD, end-stage renal disease.
